# Proteomic and Cytokine Profiling in Plasma from Patients with Normal-Tension Glaucoma and Ocular Hypertension

**DOI:** 10.1007/s10571-024-01492-3

**Published:** 2024-08-16

**Authors:** Mia Langbøl, Arevak Saruhanian, Sarkis Saruhanian, Daniel Tiedemann, Thisayini Baskaran, Rupali Vohra, Amalie Santaolalla Rives, José Moreira, Verena Prokosch, Hanhan Liu, Jan-Wilm Lackmann, Stefan Müller, Claus Henrik Nielsen, Miriam Kolko, Jens Rovelt

**Affiliations:** 1 Department of Drug Design and Pharmacology, University of Copenhagen, Jagtvej 160, Building 22, 2100 Copenhagen Ø, Denmark; 2 Department of Veterinary & Animal Sciences, University of Copenhagen, Frederiksberg, Denmark; 3 Department of Ophthalmology, Copenhagen University Hospital, Rigshospitalet-Glostrup, Glostrup, Denmark; 4 Department of Ophthalmology, Faculty of Medicine and University Hospital of Cologne, University of Cologne, 50937 Cologne, Germany; 5 CECAD/CMMC Proteomics Facility, CECAD Research Center, University of Cologne, Cologne, Germany; 6 Institute for Inflammation Research, Center for Rheumatology and Spine Diseases, Rigshospitalet, Copenhagen University Hospital, 2200 Copenhagen, Denmark; 7 Department of Odontology, Faculty of Health and Medical Sciences, University of Copenhagen, 2200 Copenhagen, Denmark

**Keywords:** Normal-tension glaucoma, Ocular hypertension, Hypoxia, Immune response, Proteomics, Cytokines

## Abstract

Primary open-angle glaucoma (POAG) is subdivided depending on eye pressure. Patients with normal-tension glaucoma (NTG) have never had high intraocular pressure (IOP) measured while patients with ocular hypertension (OHT) have high eye pressure but no signs of glaucoma. Although IOP is considered to be a risk factor for all glaucoma patients, it is reasonable to assume that other risk factors such as inflammation play a role. We aimed to characterize the proteome and cytokine profile during hypoxia in plasma from patients with NTG (*n* = 10), OHT (*n* = 10), and controls (*n* = 10). Participants were exposed to hypoxia for two hours, followed by 30 min of normoxia. Samples were taken before (“baseline”), during (“hypoxia”), and after hypoxia (“recovery”). Proteomics based on liquid chromatography coupled with mass spectrometry (LC–MS) was performed. Cytokines were measured by Luminex assays. Bioinformatic analyses indicated the involvement of complement and coagulation cascades in NTG and OHT. Regulation of high-density lipoprotein 3 (HDL3) apolipoproteins suggested that changes in cholesterol metabolism are related to OHT. Hypoxia decreased the level of tumor necrosis factor-α (TNF-α) in OHT patients compared to controls. Circulating levels of interleukin-1β (IL-1β) and C-reactive protein (CRP) were decreased in NTG patients compared to controls during hypoxia. After recovery, plasma interleukin-6 (IL-6) was upregulated in patients with NTG and OHT. Current results indicate an enhanced systemic immune response in patients with NTG and OHT, which correlates with pathogenic events in glaucoma. Apolipoproteins may have anti-inflammatory effects, enabling OHT patients to withstand inflammation and development of glaucoma despite high IOP.

**Graphical Abstract:**

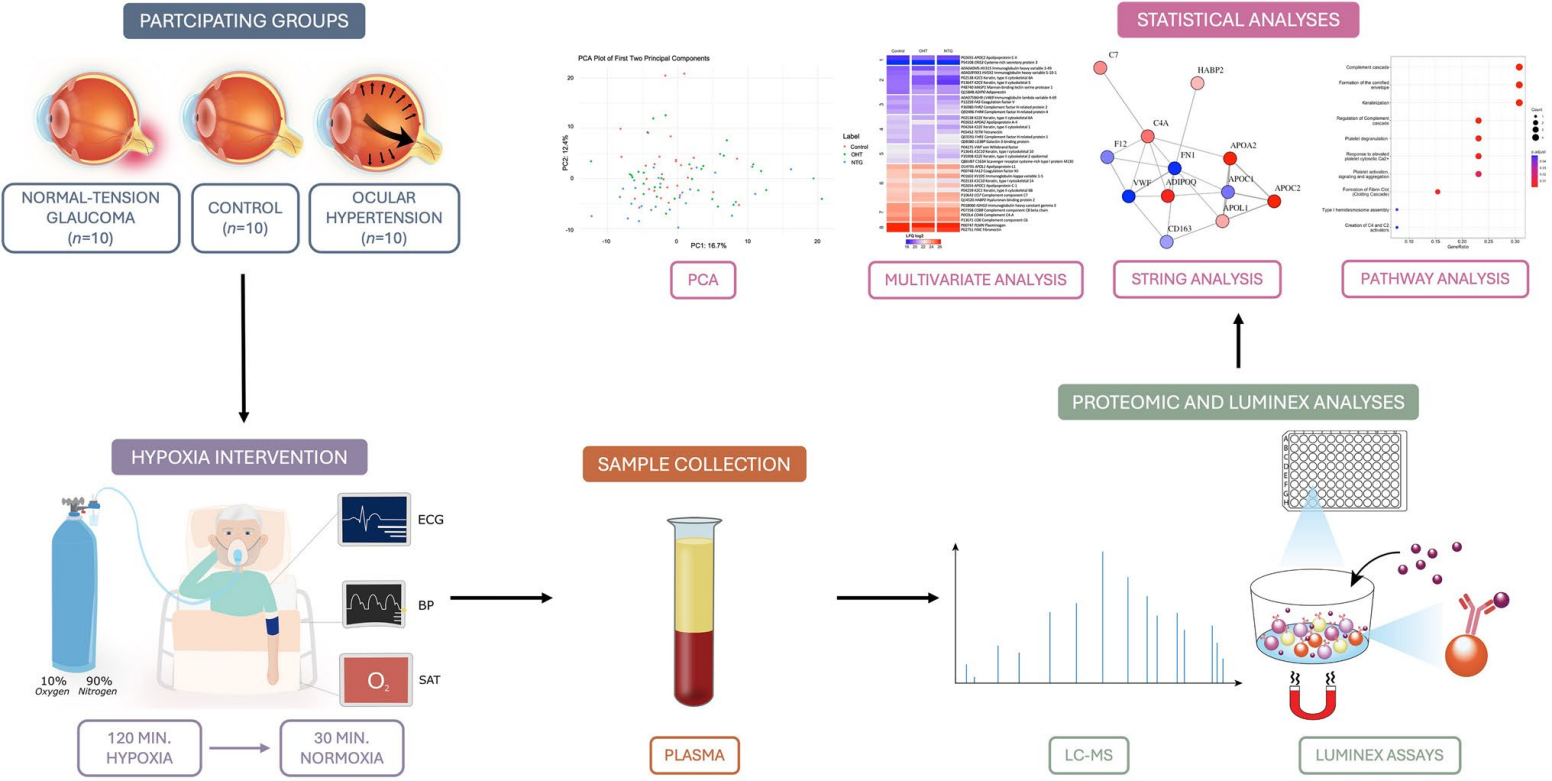

**Supplementary Information:**

The online version contains supplementary material available at 10.1007/s10571-024-01492-3.

## Introduction

Affecting nearly 76 million people, glaucoma is one of the most sight-threatening diseases worldwide (Tham et al. 2014). Despite a high prevalence, the number is expected to rise to an estimated 111 million people living with glaucoma in 2040, due to an aging population (Tham et al. 2014). Glaucoma is a spectrum of diseases that differ in pathophysiology and manifestations but share a common feature of progressive degeneration of the retinal ganglion cells (RGCs) and their axons. The progressive loss of RGCs leads to excavation of the optic nerve head, impairment of the visual field, and ultimately blindness (Gupta and Chen 2016; Schuster et al. 2020). Elevated intraocular pressure (IOP) is considered the major risk factor for glaucoma. However, about 30–40% of patients with glaucoma have an IOP within the normal range of 10–21 mmHg; a subgroup referred to as normal-tension glaucoma (NTG) (Khaw et al. 2004; Song and Caprioli 2014). Additionally, patients with ocular hypertension (OHT) have a high IOP yet they do not suffer from glaucomatous neurodegeneration. This suggests that other mechanisms beside increased IOP can cause degeneration of RGCs.

Growing evidence points to the activation of the immune response and inflammation as the main components in glaucomatous neurodegeneration (Adornetto et al. 2019; Baudouin et al. 2021; Bosco et al. 2011, 2015; Jassim et al. 2021; Wax et al. 2008; Wilson et al. 2015). It is acknowledged that glaucoma is associated with mitochondrial dysfunction and metabolic vulnerability that may lead to oxidative stress and inflammation (Jassim et al. 2021; Langbøl et al. 2023; Lascaratos et al. 2015). Pathophysiological processes seen in glaucoma, such as acute ischemia and cell damage, can lead to activation of the immune system (Dammak et al. 2023; Vohra et al. 2019). The immunological response to an injury or stress is composed of a complex network of pathways, and both the innate and adaptive immune system seem to play a central role in glaucoma (Bell et al. 2018; Jassim et al. 2021; Jiang et al. 2020; Kamat et al. 2016; Mac Nair and Nickells 2015; Rieck 2013; Russo et al. 2016; Stevens et al. 2007; Tezel 2011). The complement system is a humoral branch of the innate immune system that is composed of a complex cascade of plasma proteins to amplify the cellular immune response (Chen et al. 2022; Duarte 2021; Kamat et al. 2016; Liu et al. 2021). Studies in human and animal models of glaucoma have shown that activation of the complement system (Ahmed et al. 2004; Bonifati and Kishore 2007; Chen et al. 2022; Duarte 2021; Howell et al. 2011; Kuehn et al. 2006; Rieck 2013; Rizzo et al. 2017; Soto and Howell 2014; Stasi et al. 2006; Tezel et al. 2010), and cytokines produced by activated immune cells (Adornetto et al. 2019; Huang et al. 2010; Kondkar et al. 2018) can mediate damaging effects on neural tissue, including RGCs. Recent evidence has revealed that RGC death can be induced by various cytokines, produced from Th1 cells as well as Th2 cells, in both in vivo and in vitro models (Huang et al. 2009; Vernazza et al. 2020). Apoptosis in RGCs may be initiated by signaling pathways in the immune system and contribute to the development of glaucoma (Zeng et al. 2005). Accumulating evidence suggests that glaucoma is a systemic disease, often occurring with systemic comorbidities related to inflammation (Horwitz et al. 2016, 2017; Pache and Flammer 2006; Wandell et al. 2022). The systemic release of inflammatory cytokines and proteins may exert neurodamaging effects. This study aimed to characterize the proteome and cytokine profile in plasma from NTG and OHT patients. Participants were exposed to hypoxia to stress their vascular and immune system and thereby assess the stress response in the included groups.

## Experimental Section

The present study was an interventional study. Written informed consent was obtained from each participant for inclusion before enrolment in the study. Furthermore, the participants gave oral consent after a more thorough verbal explanation of the intervention. The study followed the tenets of the Declaration of Helsinki of 1975 with ethical approval from the National Committee on Health Research Ethics (Project identification code: H-2-2014-060).

## Power Calculation

To decide the sample size needed for the current study, a power calculation was performed on previously reported data that investigated glucose, lactate, and amino acid levels in plasma from patients with NTG and controls in the same experimental model of hypoxia (Vohra et al. 2019). In controls, lactate increased by 18% from baseline to hypoxia (based on a mean lactate level of 1.1 ± 0.16 mmol/l that was upregulated to 1.3 ± 0.9 mmol/l during hypoxia). With a power of 80% and a *p*-value of 0.05, at least ten participants should be included in each group, suggesting that the statistical power of the current study is sufficient.

## Patient Selection

A total of 30 individuals participated in the study, including 10 patients with NTG, 10 patients with OHT, and 10 age-matched healthy controls. Patients with NTG and OHT were recruited from the Department of Ophthalmology, Rigshospitalet-Glostrup, Denmark, or from general ophthalmologist. Controls were recruited either from general ophthalmologist or as spouses to patients with NTG or OHT. The recruitment process took place from January 2017 to January 2020. Over the 3 years of recruitment, 223 individuals (80 OHT patients, 78 NTG patients, and 65 controls) were identified as eligible participangts in the current study. We excluded 44 individuals (18 OHT patients, 15 NTG patients, and 11 controls) because they did not meet the inclusion and/or exclusion criteria for this particular study. After reading the information, 132 individuals (51 OHT patients, 45 NTG patients, and 36 controls) declined to participate. A total of 48 individuals (12 OHT patients, 18 NTG patients, and 18 controls) participated in the experimental setup with hypoxia. The experiment was interrupted due to altitude sickness in 5 cases (1 OHT patient, 1 NTG patient, and 3 controls). Plasma samples were withdrawn from 43 individuals (11 OHT patients, 17 NTG patients, and 15 controls). Subsequently, 6 individuals (1 OHT patient, 4 NTG patients, and 1 control) have been excluded because they no longer met the inclusion and/or exclusion criteria.

In the selection of participants, considerations were made on the recorded medical history and use of medication. Participants taking anti-inflammatory medication, that could significantly affect study results were excluded. One control took Diclofenac and one NTG patient took Avamys® while taking part in the hypoxia experiment. Diclofenac is a non-steroidal anti-inflammatory drug, that could potentially affect results. Avamys® contains adrenocortical hormone, which stimulates the release of immunosuppressive cortisol from the adrenal glands. Avamys® is administered nasally, and we assumed that its administration would not affect the results significantly. We have, however, taken the use of these medications into consideration, when interpreting results from each participant in the study.

## Inclusion and Exclusion Criteria

All patients with NTG and OHT had a full eye exam performed by a glaucoma specialist. Controls had an eye exam performed either by a glaucoma specialist or a general ophthalmologist.

### Inclusion Criteria

Inclusion criteria for patients with NTG were as follows: untreated IOP below 21 mmHg measured at different times of the day (8 a.m.–5 p.m.); open angles determined by gonioscopy, optic disc cupping; characterized by a violated ISNT rule (normal eyes show a characteristic configuration for disc rim thickness of inferior ≥ superior ≥ nasal ≥ temporal), glaucomatous visual field loss identified by Humphrey or Octopus perimetry, and significant nerve fiber loss detected by ocular coherence tomography (OCT). Inclusion criteria for patients with OHT were as follows: untreated IOP above 24 mmHg measured at different times of the day (8 a.m.–5 p.m.); open angles determined by gonioscopy; no signs of optic disc cupping; normal visual fields, and normal OCTs (Dalgaard et al. 2021; Langbøl et al. 2020, 2023; Vohra et al. 2019).

### Exclusion Criteria

Exclusion criteria for all participants were as follows: a medical history of ocular trauma, competing eye conditions other than glaucoma affecting the optic nerve; significant systemic diseases, i.e. dysregulated hypertension, heart failure, hypercholesterolemia, diabetes mellitus, autoimmune diseases, and previous cerebral infarct or hemorrhage; subjects who could not cope with the experimental setup; subjects under 50 years of age, and subjects who smoked at the time of inclusion. Medical history and list of medications were collected from each participant to check whether they met the inclusion and exclusion criteria (Langbøl et al. 2020, 2023; Vohra et al. 2019).

## Hypoxic Intervention and Sample Collection

The hypoxia model has previously been described (Dalgaard et al. 2021; Langbøl et al. 2020, 2023; Vohra et al. 2019). For a more thorough description, we refer to these publications. In brief, the participants fasted 10 h prior to the experiment. All participants were exposed to normobaric hypoxia for two hours, through a tightly fitting facemask. The mask was connected using a Y-piece to a Douglas bag, with a humidified gas mixture of 10% oxygen and 90% nitrogen. After the hypoxia period, participants were exposed to normobaric normoxia by breathing ambient air for 30 min. The experimental setup has been designed with experts in the field and medical doctors, to make sure the experiment would be safe for the participants and to minimize any discomfort. Participants were continuously monitored with a three-lead electrocardiogram and measured for heart rate, blood pressure, and arterial oxygen saturation. In case of any discomfort, the experiment was immediately interrupted. Blood samples were collected from a peripheral vein at three time points: before hypoxia (“baseline”), during hypoxia (“hypoxia”), and after 30 min of normoxia (“recovery”). At each time point, whole blood was collected in EDTA tubes and stored on ice. The blood samples were centrifuged for 10 min at 1800×*g* at 4 °C within one hour, and the supernatants were pipetted to Eppendorf tubes and stored at − 80 °C until further analyses.

## Proteomics

### Sample Preparation Overview

Albumin and immunoglobulin G (IgG) were specifically removed from the plasma samples with the ProteoPrep® Immunoaffinity Albumin and IgG Depletion Kit (PROTIA, Sigma-Aldrich, USA). Samples were processed following the manufacturer’s instruction. Samples were prepared using a modified SP3 protocol (Hughes et al. 2019) and with a small pipetting robot (Assist Plus, Integra Biosciences, USA). A sample aliquot was diluted 1:100 in SP3 buffer prior to reduction and alkylation, and the equivalent of 20 µg was used for further clean-up and digestion. After digestion, beads were removed, and samples cleaned up by SDB stage tipping (Humphrey et al. 2018) instead of described bead-based clean-up. All reagents were LC–MS grade if not indicated differently.

### Denaturation, Reduction, and Alkylation

Each well of the Armadillo 96-well plate (Thermo Scientific, USA) was filled with 47.5 μl of Red/Alk-buffer [5 mM Bond-Breaker TCEPution (Thermo Scientific), 40 mM chloroacetamide (Merck, Germany)]. Then, 2.5 μl of each sample were added to each well and mixed. The plate was sealed with a plastic seal, incubated for 5 min at 95 °C, and centrifuged for 1 min at full speed. Supernatants were transferred into a new plate.

### Bead Mixture Preparation

To prepare magnetic bead mixture, 20 μl, respectively, of Sera-Mag hydrophilic and hydrophobic SpeedBeads (Cytiva, USA) were mixed and 160 μl of destilled water (VWR, USA) were added. The tubes were placed on a magnetic rack to let the beads settle for 5 min. Afterward, supernatants were removed and discarded. The tubes were taken off the magnetic rack, washed with 200 μl of water, put back on the magnet rack, and supernatants were removed. This step was repeated two times and supernatants discarded. Finally, beads were re-suspended in 100 μl water, and stored in fridge for up to 2 weeks.

### Protein Clean-Up and Digestion

2 μl of beads mix were added to each sample. Immediately afterwards, 52 μl of 100% acetonitrile (AcN, VWR) were added to obtain a final AcN percentage of 50%. Samples were incubated for 8 min at room temperature off the magnetic rack and another 2 min on the magnetic rack. Supernatants were removed and beads washed twice with 200 μl AcN per well. Supernatants were removed after each step. After last washing step, the plate was speedvaced for 1 min to remove excess liquid. Beads were reconstituted in 25 μl digest solution (0.5 μl Trypsin (Serva, Germany), 1 µl LysC (FUJIFILM Wako Chemicals, Germany), 23.5 μl of 50 mM triethylammoniumbicarbonate (Merck). The plate was closed with a plastic seal and digested for 16 h at 25 °C. Afterwards, beads were re-suspended and acidified to a final concentration of 0.5% formic acid (FA, Merck, Germany). Beads were removed and supernatant stored at − 20 °C until analysis.

### LC–MS Analysis

Samples were analyzed by the CECAD Proteomics Facility on an Orbitrap Exploris 480 (Thermo Scientific, granted by the German Research Foundation under INST 1856/71-1 FUGG) mass spectrometer equipped with a FAIMS pro differential ion mobility device that was coupled to an UltiMate 3000 (Thermo Scientific). Samples were loaded onto a precolumn (Acclaim 5 µm PepMap 300 cartridge, Thermo Scientific) for 1 min at 15 µl/min before reverse-flushed onto an in-house packed analytical column (15 cm length, 150 µm inner diameter, filled with 2.7 µm Poroshell EC120 C18, Agilent, USA). Peptides were chromatographically separated at a constant flow rate of 1 µl/min running eluent A (0.1% FA) against eluent B (0.1% FA in 80% AcN) and the following gradient: initial 6% B, up to 35% B in 35 min, up to 55% B within 3 min and up to 95% solvent B within 1.0 min, followed by column wash with 95% solvent B and reequilibration to initial condition. The FAIMS pro was operated at − 50 V compensation voltage and electrode temperatures of 99.5 °C for the inner and 85 °C for the outer electrode.

MS1 scans were acquired from 380 to 900 m/z at 15 k resolution. Maximum injection time was set to 22 ms and the advanced gain control (AGC) target to 100%. MS2 scans ranged from 400 to 880 m/z and were acquired at 15 k resolution with a maximum injection time of 22 ms and an AGC target of 100% and optimized window placement activated. MS2 scans were acquired in 30 windows with a constant width of 16 m/z windows. After the first round of MS2 acquisitions, a second round of MS2 acqusitions was run with the same 16 m/z windows size, but all windows were offset by 8 m/z, resulting in a range of 392 m/z to 888 m/z, following the concept as presented by Ludwig et al. (2018). Both acquisitions were afterwards used for deconvolution using ProteoWizard, resulting in effective 8 m/z windows for data analysis (Chambers et al. 2012). All scans were stored as centroid.

### Simulation of Spectral Library

A human canonical Uniprot reference proteome fasta file (UP5640, downloaded at: 26.08.2020) was converted to a Prosit upload file with the convert tool in encyclopedia 0.9.5 (Searle et al. 2018) using default settings: Trypsin, up to 1 missed cleavage, range 380–900 m/z, charge states 2+ and 3+, default charge state 3 and NCE 33. The csv file was uploaded to the Prosit webserver and converted to a spectrum library in generic text format (Gessulat et al. 2019). The resulting library containing 28,325 protein groups was used as the base library for data analysis in DIA-NN 1.7.16 (Demichev et al. 2020).

### Sample Processing in DIA-NN

Samples were analyzed with the generated Prosit library (Gessulat et al. 2019) as base and the match-between-runs function enabled in DIA-NN (Demichev et al. 2020). Here, samples are directly used to refine the library for a second search of the sample data. DIA-NN was run with the additional command line prompt “—report-lib-info”. Further output settings were: filtered at 0.01 false discovery rate (FDR), N-terminal methionine excision enabled, maximum number of missed cleavages set to 1 minimum peptide length set to 7, maximum peptide length set to 30, minimum precursor m/z set to 380, maximum precursor m/z set to 900, cysteine carbamidomethylation enabled as a fixed modification. Afterwards, DIA-NN output was further filtered on library *q*-value and global *q*-value ≤ 0.01 using R (version 3.6.3). Finally, label-free quantification (LFQ) values calculated using the DIA-NN R-package.

### Statistical Analyses of Proteomics

Afterwards, analysis of results was performed in Perseus 1.6.15 (Tyanova et al. 2016). LFQ values for each protein group were log2 transformed for better visibility and initial smoothing. Afterwards, everything was filtered on 70% data completeness in at least one of the groups of interest. Bioinformatical analyses were performed in R (version 4.3.2.) (R Core Team 2022). We used the R-package Linear Models for Microarray Data (limma) to analyze differential expression between phenotypes and timepoints using unimputed data (R Core Team 2022; Ritchie et al. 2015). The limma method involves fitting a linear model to the expression data for each protein, and then applying empirical Bayes moderation to the standard errors. Differential expression was assessed by calculating moderated *t*-statistics for each protein. Multiple testing correction was performed using the Benjamini–Hochberg method to control the FDR. Further analyses were carried out on differentially expressed proteins with an FDR < 0.05. We have reported protein fold change (FC) as log2FC and adjusted *p*-values. Statistical testing revealed no differences between time points. Therefore, measurements from each time point were pooled to further increase the *n* and statistical power of the study. If there is an interaction between phenotype and time point, pooling can introduce confounding factors. The observed difference in pooled data therefore might not be purely due to phenotype but could be influenced by how the phenotypes change over time. We have tested this and found no interaction.

KEGG and Reactome pathway analyses were performed with the library clusterProfiler. A *p*-value of *p* < 0.05 was used as cutoff for KEGG and Reactome pathway analyses. The *p*-values were adjusted for multiple testing using the FDR method. To construct protein–protein interaction networks, STRING analyses were performed in the online StringDB (version 12.0, accessed on 18 December 2023) (Szklarczyk et al. 2023) and visualized in R with iGraph (R Core Team 2022). For the STRING analyses, we included all interaction types available in the database. These include known interactions from curated databases, experimentally determined interactions, predicted interactions based on gene neighborhood, gene fusions, and gene co-occurrence, as well as interactions inferred from text mining, co-expression, and protein homology. The default medium confidence score threshold of 0.4 was applied to ensure that interactions with a reasonable level of confidence were included in the network.

## Luminex Assays

Plasma levels of eight cytokines were measured by combining selected cytokines in a multiplexing strategy using the Bio-Plex Pro Human Cytokine 8-Plex Screening Panel consisting of the reagent kit 3 (BIO-RAD, USA; #171304090M), the panel standard (BIO-RAD, USA, # 12007919), and bead sets for the included cytokines interleukin-1β (IL-1β, #171B5001M), interleukin-4 (IL-4, #171B5004M), interleukin-6 (IL-6, #171B5006M), interleukin-10 (IL-10, #171B5010M), interleukin-17 (IL-17, #171B5014M), interferon-α (IFN-α, #171B6010M), interferon-γ (IFN-γ, #171B5019M), and tumor necrosis factor-α (TNF-α, #171B5026M). Samples were diluted 1:2. Additionally, TGF-β1 was measured in a single plex setup consisting of the reagent kit 3 (BIO-RAD, USA; #171304090M), the TGF-β1 standard (BIO-RAD, USA, #171X40001), and the bead and detection antibody set (BIO-RAD, USA, #171W4001M). Prior to TGF-β1 analyses samples were treated with 1N HCl and 1,2N NaOH. Samples for TGF-β1 analyses were diluted 1:16. CRP was measured with the Magnetic Luminex® Performance Assay Human C-Reactive Protein/CRP Kit (R&D Systems, United Kingdom, #LOBM1707). Samples for CRP analyses were diluted 1:250. The assays were run on a Luminex-200 platform (Luminex Corporation, Austin, TX) following the manufacturer’s instructions.

For some targets, signal detection was too low. The lower limits of detection (LOD) were 0.24 pg/ml for IL-1β, 0.09 pg/ml for IL-4, 0.34 pg/ml for IL-6, 0.69 pg/ml for IL-10, 1.16 pg/ml for IL-17, 0.46 pg/ml for IFN-α, 1.05 pg/ml for IFN-γ, 1.13 pg/ml for TNF-α, 1.69 pg/ml for TGF-β1, and 1.4 pg/ml for CRP. If < 50% of the samples were measurable the target was excluded from the study. The excluded targets were IL-4, IL-10, and IL-17.

### Statistical Analyses of Cytokines and CRP

Data were analyzed with GraphPad Prism 10 software (GraphPad Software, version 10.0.2, San Diego, CA, USA). *p* < 0.05 was considered statistically significant. Comparisons between groups were made using Mann–Whitney *U*-test and comparisons between time points were made using Wilcoxon signed-rank test. Outliers were identified using the ROUT method with *Q* = 1%.

## Results

### Demographics and Ophthalmological Characteristics

Demographic data of age, height, weight, and sex were collected on the day of enrollment (Table 1). All participants went through and eye exam to perform a strict phenotyping, including IOP and visual field tests (Table 1). As anticipated, IOP was higher on both eyes in OHT patients compared to both NTG patients and controls, while MD was higher on both eyes in NTG patients compared to both OHT patients and controls. No significant differences were identified between the groups concerning age, BMI, and sex (Table 1).

**Table 1:** Demographic and ophthalmic data on participants

	Control	NTG	OHT
*N*	10	10	10
Age (years)	67 ± 8.1	73 ± 6.8	72 ± 4.3
BMI^a^	27.3 ± 7.1	24.0 ± 2.6	25.3 ± 2.4
Sex (M/W)	7/3	6/4	5/5
IOP OD (mmHg)	13.6 ± 1.8****	12.1 ± 2.0****	29.3 ± 5.7
IOP OS (mmHg)	13.6 ± 2.0****	12.5 ± 2.4****	31.2 ± 5.5
MD OD (dB)	0.9 ± 1.6^^	7.2 ± 7.0	1.4 ± 1.7^
MD OS (dB)	1.0 ± 2.6^^^^	11.1 ± 6.4	0.9 ± 1.4^^^^

Data are represented as mean values ± SD

*BMI* body mass index, *IOP* intraocular pressure, *M* men, *MD* mean defect, *N* number of participants, *NTG* normal-tension glaucoma, *OD* oculus dexter, *OHT* ocular hypertension, *OS* oculus sinister, *SD* standard deviation, *W* women

****p < 0.0001 (different from OHT patients). ^^^^*p* < 0.0001; ^^*p* < 0.01; ^*p* < 0.05 (different from NTG patients). Published in Langbøl et al. (2023)

^a^$$\text{BMI}=\frac{\text{Weight} (\text{kg})}{{\text{Height} (\text{m})}^{2}}$$

### Stress Response of Systemic Vital Parameters

Heart rate, oxygen saturation, partial pressure of oxygen, and partial pressure of carbon dioxide were observed, with no differences between the three study groups, indicating a similar response to hypoxic intervention. Observations of systemic vital parameters have been presented by Langbøl et al. (2020).

### Proteomics

#### Protein Detection and Clustering

**Fig. 1:**
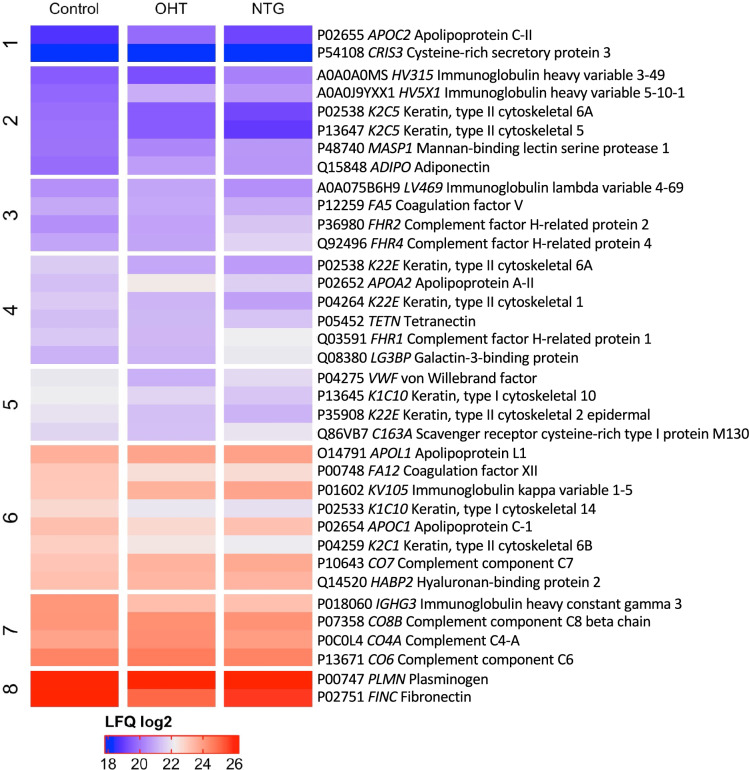
Heatmap showing the differential expression of proteins identified in plasma from patients with NTG (*n* = 10), OHT (*n* = 10), and controls (*n* = 10). Data in each group is pooled from the three time points (“baseline”, “hypoxia”, and “recovery”). Clustering is based on the control group and protein names are displayed as row labels. Up-regulated protein expression is displayed in red, downregulated expression in blue, and intermediate-regulated expression is in shades of red and blue. LFQ values refer to mean values. *LFQ* label-free quantification

**Fig. 2:**
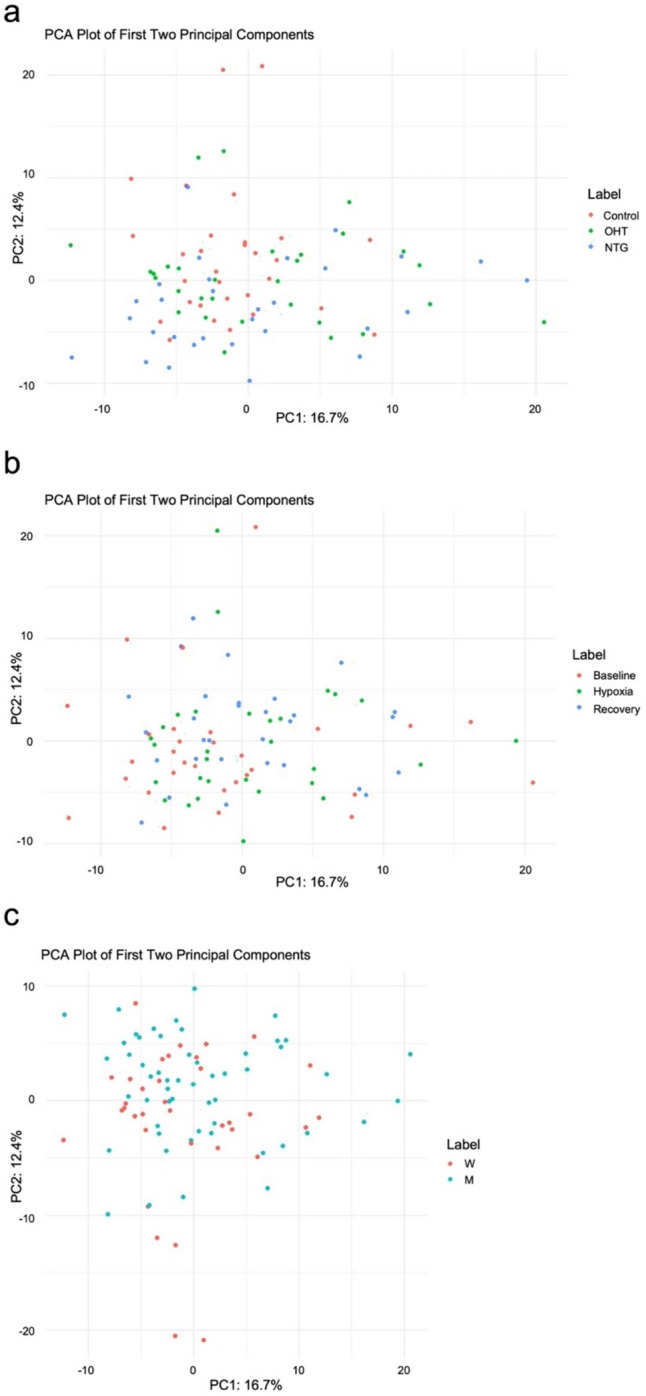
PCA score plots of the full dataset. PCA score plot of samples colored according to **a** test group (controls, OHT, and NTG patients), **b** time point (“baseline”, “hypoxia”, and “recovery”), and **c** sex (men and women). *M* men; *NTG* normal-tension glaucoma; *OHT* ocular hypertension; *PCA* principal component analysis; *W* women

Proteomic analysis yielded the identification of 282 proteins. Of these, 35 were differentially expressed in NTG, OHT, and control subjects (Fig. 1). Principal component analysis (PCA) revealed no clear clustering between test groups, time points, or sexes (Fig. 2a–c). No statistically significant differences were observed between time points as a response to hypoxia in any of the included groups, nor between groups at any of the time points.

Multivariate analyses of differences between phenotypes yielded a total of 19 plasma proteins that were significantly associated with NTG and 14 plasma proteins that were significantly associated with OHT compared to controls. Twelve plasma proteins were significantly different between patients with NTG and OHT. To study the relative abundances of the differentially expressed proteins, the FC in protein levels between the three groups was determined accompanied by an adjusted *p*-value of the change (Table 2). Eight keratins were differentially expressed between patients with NTG and controls. They were not included in the further analyses, because they most likely represent contaminants from sample handling.

**Table 2:** Differentially expressed proteins, grouped by compared groups and sorted by descending log2FC

UniProt ID	Gene symbol	Protein name	log2FC*	Adjusted *p*-value
Patients with NTG compared to controls				
Q08380	*LGALS3BP*	Galectin-3-binding protein	0.668	0.0171
P36980	*CFHR2*	Complement factor H-related protein 2	0.626	0.00880
P01602	*IGKV1-5*	Immunoglobulin kappa variable 1-5	0.570	0.0331
Q92496	*CFHR4*	Complement factor H-related protein 4	0.480	0.0332
P10643	*C7*	Complement component C7	0.366	0.0459
P48740	*MASP1*	Manna-binding lectin serine protease 1	0.289	0.0408
P05452	*CLEC3B*	Tetranectin	− 0.220	0.0481
P12259	*F5*	Coagulation factor V	− 0.249	0.0408
P54108	*CRISP3*	Cysteine-rich secretory protein 3	− 0.461	0.0331
P00748	*F12*	Coagulation factor XII	− 0.732	0.000166
P01860	*IGHG3*	Immunoglobulin heavy constant gamma 3	− 1.0762	0.000361
Patients with OHT compared to controls				
A0A0J9YXX1	*HV5X1*	Immunoglobulin heavy variable 5-10-1	1.220	0.0175
P02655	*APOC2*	Apolipoprotein C-II	0.929	0.0406
P02652	*APOA2*	Apolipoprotein A-II	0.900	0.00350
Q15848	*ADIPOQ*	Adiponectin	0.798	0.00144
P0C0L4	*C4A*	Complement C4-A	0.495	0.0475
P10643	*C7*	Complement component C7	0.389	0.0365
O14791	*APOL1*	Apolipoprotein L1	0.283	0.0365
Q14520	*HABP2*	Hyaluronan-binding protein 2	0.209	0.00496
Q86VB7	*CD163*	Scavenger receptor cysteine-rich type 1 protein M130	− 0.398	0.0406
P00748	*F12*	Coagulation factor XII	− 0.531	0.0108
P02654	*APOC1*	Apolipoprotein C-I	− 0.557	0.0221
P01860	*IGHG3*	Immunoglobulin heavy constant gamma 3	− 0.803	0.0153
P02751	*FN1*	Fibronectin	− 0.942	0.00496
P04275	*VWF*	Von Willebrand factor	− 1.0185	0.0166
Patients with OHT compared to NTG				
P02655	*APOC2*	Apolipoprotein C-II	0.967	0.0257
P02652	*APOA2*	Apolipoprotein A-II	0.950	0.00152
A0A075B6H9	*IGLV4-69*	Immunoglobulin lambda variable 4-69	0.687	0.00898
P0C0L4	*C4A*	Complement C4-A	0.569	0.0137
P13671	*C6*	Complement component C6	0.353	0.00152
P07358	*C8B*	Complement component C8 beta chain	0.282	0.00898
P00747	*PLG*	Plasminogen	0.190	0.00904
Q14520	*HABP2*	Hyaluronan-binding protein 2	0.178	0.0161
Q92496	*CFHR4*	Complement factor H-related protein 4	− 0.571	0.00898
A0A0A0MS15	*IGHV3-49*	Immunoglobulin heavy variable 3-49	− 0.620	0.0145
Q08380	*LGALS3BP*	Galectin-3-binding protein	− 0.665	0.0114
Q03591	*CFHR1*	Complement factor H-related protein 1	− 0.727	0.00484

*FC* fold change

*Indicates up- or downregulation in the first-mentioned group

#### KEGG and Reactome Pathway Analysis

To check if certain pathways were differentially regulated between groups, KEGG and Reactome pathway analysis was performed. Results of the KEGG and Reactome pathway analyses are presented in Supplementary Figs. S1 and S2 as online resource. KEGG pathway analysis indicated a significant involvement of “complement and coagulation cascades” in both NTG (GeneRatio: 6/6, *p*.adjust = 4.83^−12^) and OHT (GeneRatio: 4/10, *p*.adjust = 5.10^−5^) patients compared to controls. The same pathways were related when comparing NTG and OHT patients (“Complement and coagulation cascades”: GeneRatio: 4/10, *p*.adjust = 5.10^−5^). Reactome pathway analysis confirmed the involvement of the “complement cascade” in patients with NTG (GeneRatio: 4/9, *p*.adjust = 2.23^−6^) and OHT (GeneRatio: 2/11, *p*.adjust = 3.69^−3^) compared to controls.

KEGG pathway analysis indicated that several hemostasis-related pathways were related to patients with NTG (including “Platelet degranulation”: GeneRatio: 3/9, *p*.adjust = 9.13^−4^; “Platelet activation, signaling and aggregation”: GeneRatio: 3/9, *p*.adjust = 4.41^−3^; “Formation of Fibrin Clot (Clotting Cascade)”: GeneRatio: 2/9, *p*.adjust = 1.00^−3^) and OHT (including “Intrinsic Pathway of Fibrin Clot Formation”: GeneRatio: 2/11, *p*.adjust = 1.44^−3^; “Integrin signaling”: GeneRatio: 2/11, *p*.adjust = 1.84^−3^; “Formation of Fibrin Clot (Clotting Cascade)”: GeneRatio: 2/11, *p*.adjust = 2.60^−3^) compared to controls. When comparing NTG and OHT patients, hemostasis-related pathways were also related (including “Intrinsic Pathway of Fibrin Clot Formation”: GeneRatio: 2/11, *p*.adjust = 1.44^−3^; “Integrin signaling”: GeneRatio: 2/11, *p*.adjust = 1.84^−3^; “Formation of Fibrin Clot (Clotting Cascade)”: GeneRatio: 2/11, *p*.adjust = 2.60^−3^).

In OHT patients, KEGG pathway analysis indicated a relation to “cholesterol metabolism” (GeneRatio: 3/10, *p*.adjust = 3.14^−5^) in comparison with controls. Comparing NTG and OHT patients confirmed the association (“Cholesterol metabolism”: GeneRatio: 3/10, *p*.adjust = 3.14^−5^). Reactome pathway analysis also supported the involvement of proteins related to cholesterol metabolism in OHT patients compared to both controls and NTG patients (including “Plasma lipoprotein assembly”: GeneRatio: 3/11, *p*.adjust = 5.42^−5^; “Chylomicron assembly”: GeneRatio: 2/11, *p*.adjust = 6.10^−4^; “Chylomicron remodeling”: GeneRatio: 2/11, *p*.adjust = 6.10^−4^).

#### STRING Network Analysis

**Fig. 3:**
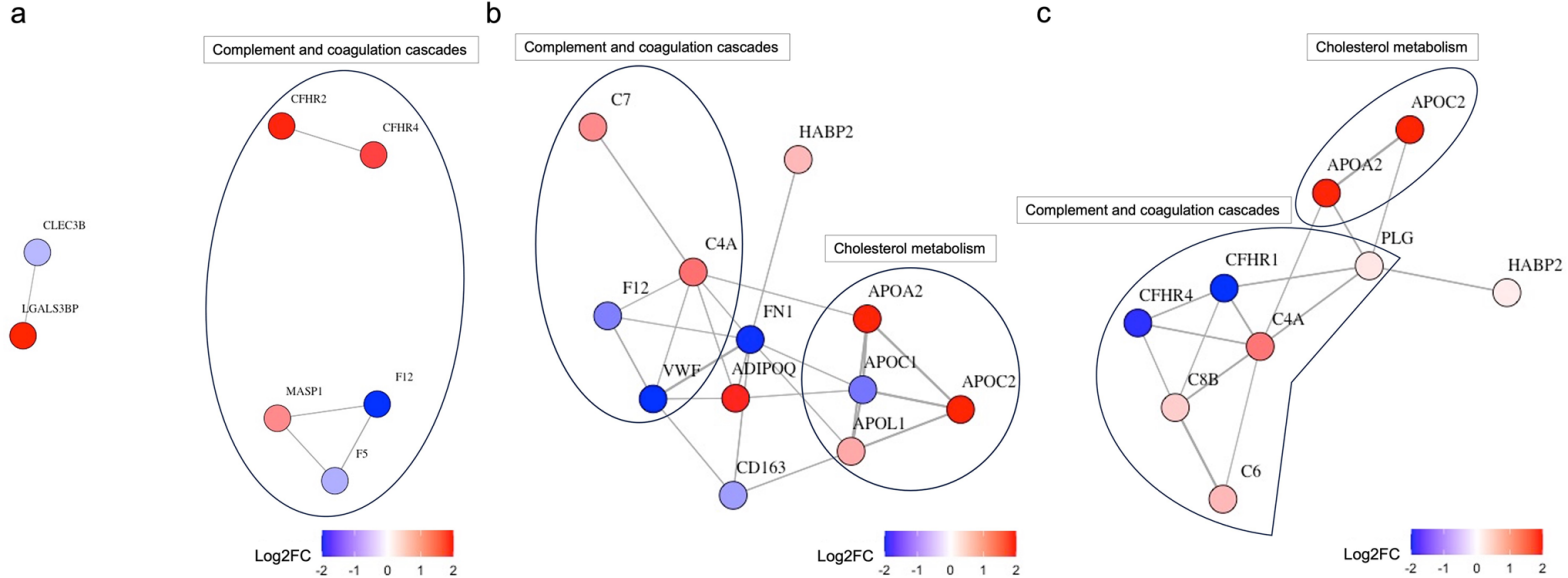
STRING network analysis of differentially expressed proteins in plasma from **a** patients with NTG compared to controls, **b** patients with OHT compared to controls, and **c** patients with NTG compared to patients with OHT. Each protein is represented as a node, and lines represent interactions between two nodes. Red nodes indicate more abundant proteins and blue nodes indicate less abundant proteins. Proteins are separated based on their involvement in biological pathways according to KEGG analysis

STRING analysis was used to generate a protein–protein interaction network based on known interactions (Fig. 3a–c). The three networks are highly enriched in proteins from the complement and coagulation cascades. Additionally, proteins involved in cholesterol metabolism are enriched in patients with OHT that interact at several points with proteins from the complement and coagulation cascades.

### Cytokines and CRP

To assess the immune response of the included groups to hypoxic intervention, the pro-inflammatory cytokines IL-1β, TNF-α, IL-6, IL-17, IFN-α, and IFN-γ as well as the anti-inflammatory cytokines TGF-β1, IL-4, and IL-10 were measured in plasma. IL-4, IL-10, and IL-17 were below the LOD level and excluded from the study. Additionally, C-reactive protein (CRP) was measured as a marker for systemic inflammation.

**Fig. 4:**
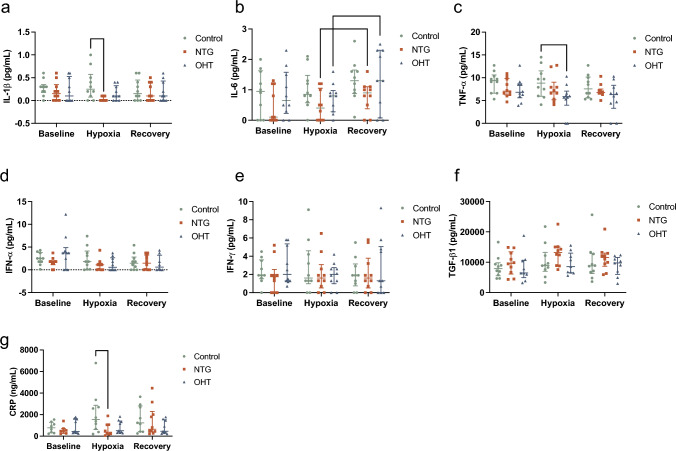
Plasma levels of cytokines and CRP in patients with NTG (*n* = 10) and OHT (*n* = 10) compared to controls (*n* = 10) at baseline, hypoxia, and recovery. Plasma levels of **a** IL-1β, **b** IL-6, **c** TNF-α, **d** IFN-α, **e** IFN-γ, **f** TGF-β1, and **g** CRP. Mann–Whitney *U*-test was used to test differences between groups and Wilcoxon-signed rank test to test differences between time points. Data are presented as individual data points with median and the interquartile range. *p < 0.05. *CRP* C-reactive protein; *IFN-α* interferon-α; *IFN-γ* interferon-γ; *IL-1β* interleukin-1β; *IL-6* interleukin-6; *TGF-β1* transforming growth factor-β1; *TNF-α* tumor necrosis factor-α

Circulating IL-1β and CRP levels were decreased in NTG patients compared to controls during hypoxia (Fig. 4a: *p* = 0.0105 and Fig. 4g: *p* = 0.0343). IL-6 increased in the recovery period from hypoxia in patients with NTG and OHT (Fig. 4b: *p* = 0.0312 and *p* = 0.0391, respectively). Also, during hypoxia, TNF-α was decreased in OHT patients compared to controls (Fig. 4c: *p* = 0.0492). No significant differences were observed in IFN-α, IFN-γ, and TGF-β1 (Fig. 4d–f).

Moreover, CRP levels were decreased in NTG patients compared to controls during hypoxia (Fig. 4g: *p* = 0.0343).

## Discussion

This study was conducted to better understand the proteomic and cytokine profile in plasma from patients with NTG and OHT compared to healthy control subjects. Furthermore, we sought to discover alterations during hypoxic stress intervention. An experimental human model that stresses the vascular and metabolic system of participants by exposing them to hypoxia followed by a recovery period was therefore developed (Dalgaard et al. 2021; Langbøl et al. 2020, 2023; Vohra et al. 2019).

In recent years, studies have indicated that changes in the immunological response is correlated with the pathogenesis of glaucoma (Huang et al. 2010; Vohra et al. 2013). The present study is to our knowledge the first to report on proteomics and cytokine changes in plasma from NTG and OHT. Former studies have mainly carried out proteomics analyses on aqueous humor samples from glaucoma patients (Adav et al. 2018; Chowdhury et al. 2010; Duan et al. 2010; Kliuchnikova et al. 2016; Kodeboyina et al. 2020; Lee et al. 2022; Liu et al. 2022). One study by González-Iglesias et al. have performed proteomics on serum samples from patients with primary open-angle glaucoma (POAG) and pseudoexfoliation glaucoma compared to healthy controls by ProteoMiner™, two-dimensional fluorescent difference gel electrophoresis (2D-DIGE), MALDI-TOF/TOF, and nanoLC-MS–MS. It is important to perform protein analyses on plasma samples from glaucoma patients to grasp the signature of glaucoma in the systemic proteome. However, investigations of protein in plasma from patients with NTG and OHT are limited. The current investigations may help guide future studies to discover protein biomarkers and targets for treatment.

## Immune System and Hemostasis

Proteomic profiling revealed differential expression of complement- and hemostasis-related proteins supported by KEGG and Reactome pathway analyses. Growing evidence indicates that dysregulation of the complement system is involved in glaucoma and early stages of glaucoma have been characterized by upregulation and activation of complement cascade proteins (Adav et al. 2018; Johnson et al. 2000; Stevens et al. 2007). Proteins involved in the complement cascade including component C4-A (C4A), component C6 (C6), component C7 (C7), component C8 beta chain (C8B), and mannan-binding lectin serine protease 1 (MASP1) differed significantly between the groups. C4A differed between patients with OHT and both controls and patients with NTG, while C6 and C8B differed only between OHT and NTG patients. C7 differed between both patients with NTG and OHT compared to controls and MASP1 differed between patients with NTG and controls. The same was true for complement factor H-related proteins [1 (CFHR1), 2 (CFHR2), and 4 (CFHR4)], which have roles in regulation of the complement system (Skerka et al. 2013). Differential expression of proteins related to the complement system seems to play a role in both NTG and OHT. CRP is a part of the complement system and an acute phase protein made by the liver. CRP has been shown to increase in plasma from NTG patients (Leibovitch et al. 2005). Our study did not confirm such findings. In healthy adults, a CRP level below 3000 ng/ml is considered normal while levels between 3000 and 10,000 ng/ml indicate low-grade inflammation and levels above 10,000 ng/ml indicate pronounced inflammation (Nehring et al. 2023). The CRP levels of the included groups in this study indicate that none of them have low-grade inflammation or overt inflammation.

Additionally, other immune-related proteins were differentially expressed between the groups. Antibodies are immunoglobulins, and IgG1 and IgG3 are involved in the induction of phagocytosis and antibody-dependent cellular cytotoxicity (ADCC), as well as in the activation of the classical pathway of the complement system, together with IgM (Adav et al. 2018; Chen et al. 2022). Though albumin and IgG were depleted from our samples, five immunoglobulin regions were differentially expressed in patients with NTG and OHT including immunoglobulin heavy constant gamma 3 (marker for IgG), two variable regions of immunoglobulin light chains (kappa and lambda), and two variable regions of immunoglobulin heavy chains. This is probably explained by the high but limited efficiency of the depletion technique.

Adiponectin which is a hormone with anti-inflammatory properties was related to OHT (Bazan 2018; Natto et al. 2019). Cysteine-rich secretory protein 3 (CRISP3), which was related to NTG, has been proposed to be involved in the innate immune system and chronic inflammation (Belardin et al. 2019; Udby et al. 2004). Scavenger receptor cysteine-rich type 1 protein M130 (CD163), which was related to OHT, and galectin-3 binding protein (LGALS3BP), which was related to NTG, both belong to the scavenger receptor cysteine-rich family and have been proposed to play a role in the innate immune system (Capone et al. 2021; Loimaranta et al. 2018; Van Gorp et al. 2010). Furthermore, LGALS3BP binds galectin-3, which has been suggested to be associated with glaucoma (Margeta et al. 2022).

Compared to controls, more inflammatory proteins were differentially regulated during hypoxia in patients with NTG than in patients with OHT, which may indicate a stronger immune response. These results are in accordance with previous studies suggesting the central role of the immune response in the pathogenesis of glaucoma (Adornetto et al. 2019; Bosco et al. 2011, 2015; Jassim et al. 2021; Wax et al. 2008; Wilson et al. 2015). Activation of the immune system, including complement activation, may cause neurodegeneration and RGC loss in glaucoma patients, and therapeutic inhibition of the complement system potentially holds a place in the treatment of glaucoma (Gassel et al. 2020; Liu et al. 2021). These results indicate that the immune system is activated in patients with OHT, but not to the same extent as in patients with NTG. Patients with OHT do not suffer from neurodegeneration but are at higher risk of developing glaucoma (Gordon et al. 2002; Van Gestel et al. 2015). These results indicate that OHT is an early stage of glaucoma, or that OHT patients have a more tight regulation of the immune response, which may protect RGCs from inflammation-induced damage.

The coagulation cascade plays a major role in hemostasis and is rapidly activated upon injury or stress, to stop bleeding and prevent invasion of microorganisms (Amara et al. 2008; Pryzdial et al. 2022). In the current study, coagulation factors were downregulated, both in patients with NTG [coagulation factor V (F5) and factor XII (F12)] and in patients with OHT (F12), compared to controls. Coagulation proteins interact with other processes including inflammation (Amara et al. 2008; Pryzdial et al. 2022). In this regard, proteins of the complement system and coagulation cascade interact at several points (Afshar-Kharghan 2017) supported by our STRING analyses. The lower expression of F5 in NTG patients and plasminogen in OHT patients might indicate activation of fibrinolysis due to hemorrhage (Amara et al. 2008). Plasminogen is also related to inflammatory processes by interacting with several complement proteins (Baker and Strickland 2020). Hyaluronan-binding protein 2 (HABP2), which was related to OHT, is an extracellular serine protease involved in the extrinsic pathway of blood coagulation by activation of F12 and fibrinolysis by activation of prourokinase type plasminogen activator (Choi-Miura et al. 1996; Kanse et al. 2008; Mambetsariev et al. 2010; Römisch 2002). Tetranectin, fibronectin, and Von Willebrand Factor, which were related to NTG or OHT, have also been suggested to be involved in hemostasis (Desch 2018; Liu et al. 2022; Moser et al. 1993). Adiponectin and fibronectin were not directly associated with the complement and coagulation cascade in bioinformatic analyses, but the literature suggest their involvement in both the immune response and hemostasis (Bazan 2018; Mirzaei et al. 2017; Natto et al. 2019; Poli et al. 2004; Upadhyay 2015). Plasma levels of hemostatic regulators were, in general, downregulated in NTG and OHT patients, indicating an impaired coagulation cascade in these patients. Though pathways of the immune system and hemostasis seem related to both NTG and OHT, bioinformatic comparisons of the two groups indicate that complement and coagulation pathways are regulated differently, which could mean that different branches of the pathways are affected in the two conditions.

## Apolipoproteins in OHT Patients

Apolipoproteins are important in lipid metabolism, especially in the transport and redistribution of lipids between cells and tissues (Liu et al. 2021; Mahley et al. 1984). Three apolipoproteins, including A-II (APOA2), C-II (APOC2), and L1 (APOL1), were upregulated in patients with OHT compared to controls (APOA2, APOC2, and APOL1) and NTG patients (APOA2 and APOC2). Apolipoprotein C-I (APOC1) was downregulated in OHT patients compared to controls. The KEGG and Reactome pathway analyses performed suggest the involvement of cholesterol metabolism and related pathways in OHT. The composition of apolipoprotein and high-density lipoprotein (HDL) affects the properties of the lipoprotein and can influence the immune response (Furlaneto et al. 2002). In line with this, STRING analysis suggested several interactions between apolipoproteins and proteins involved in the complement and coagulation cascades. APOA2, APOC1, APOC2, and APOL1 have been positively correlated with antioxidant capacity (Boisfer et al. 2002; Davidson et al. 2009; Fuior and Gafencu 2019; Swertfeger et al. 2017). They are all components of HDL3, indicating that this specific HDL lipoprotein may play a particular role in OHT (Brites et al. 2017; Davidson et al. 2009). HDL3 has been proposed to play a role in cell death and innate immunity (Pérez-Morga et al. 2005; Wan et al. 2008). HDL3 is also protective in the vascular endothelium by its antioxidant, anti-inflammatory, and antithrombotic effects (Camont et al. 2011; Davidson et al. 2009; Nusinovici et al. 2022; Pirillo et al. 2013). Several studies have suggested that high blood levels of HDL3 is associated with a lower risk of glaucoma (Nusinovici et al. 2022). Low levels of HDL3 in glaucoma patients may cause increased levels of cholesterol and atherosclerosis in retinal tissues that could reduce ocular blood flow and cause RGC loss (Nusinovici et al. 2022; Resch et al. 2009).

High levels of apolipoproteins related to HDL3 in OHT patients may optimize the transport of cholesterol back to the liver for excretion through reverse cholesterol transport (RCT) (Nusinovici et al. 2022). We hypothesize that the differentially expressed apolipoproteins identified have protective anti-inflammatory and antioxidant properties that enable these individuals to withstand development of glaucoma despite high IOP. These findings are in concordance with evidence from our previous studies, indicating that lipid mediators and metabolites are increased and play an important protective role in OHT through their suggested antioxidant capacity (Langbøl et al. 2020, 2023). Thus, HDL3 cholesterol metabolism may be a potential target for novel treatment strategies in glaucoma. No previous studies have suggested this role of these apolipoproteins, and it should be validated in future studies.

## Pro-inflammatory Cytokines and CRP

Since characterization of the proteome showed involvement of the complement system and other proteins of the immune response, we performed analyses of selected cytokines and the acute phase protein CRP to further elucidate the role of inflammation in NTG and OHT. The results indicate that mainly pro-inflammatory cytokines of the innate immune system are involved in the pathogeneses of NTG and OHT. IL-1β and CRP were decreased in NTG patients compared to controls after two hours of hypoxia, indicating that NTG patients fail to trigger the increase of pro-inflammatory signals that initiate the appropriate immune response to stress (Leibovitch et al. 2005). It could be due to a low production or an inability to release the proteins from cells.

Plasma levels of IL-6 increased from hypoxia to recovery in patients with NTG and OHT, which indicates initiation of an acute immune response. IL-6 is important for the production of acute phase proteins and other pro-inflammatory cytokines (Benitez-del-Castillo et al. 2019; Leibovitch et al. 2005). The increase in expression of IL-6 from hypoxia to recovery in patients with NTG and OHT indicates that upregulation of the remaining cytokines and CRP happen after termination of the experiment. A prolonged hypoxia or recovery period might have revealed more changes in cytokine levels; it is possible that some of these changes are only evident after days or even weeks. The lacking upregulation of IL-6 in controls additionally indicates that patients with NTG and OHT are more vulnerable to hypoxic stress intervention.

Exposure to hypoxia induced regulation of TNF-α that resulted in decreased levels in patients with OHT compared to controls. These results may indicate that OHT patients modulate their immune response to avoid excessive activation of the immune system that could otherwise have detrimental side effects besides defeating inflammation. TNF-α has been widely associated with glaucoma and RGC loss (Dammak et al. 2023; Khalef et al. 2017; Kitaoka et al. 2006; Kondkar et al. 2018; Liang et al. 2007; Nakazawa et al. 2006; Tezel et al. 2001). These results may help explain why OHT patients do not suffer from loss of RGCs, despite having the most common risk factor of glaucoma, elevated IOP. In that case, TNF-α blockers might be effective in the treatment of glaucoma. Genetic or pharmacological depletion of TNF-α or its receptors has been shown to stimulate RGC survival (Kondkar et al. 2018; Nakazawa et al. 2006; Tezel et al. 2004). Increasing evidence suggest that patients with OHT can resist development of glaucoma (Langbøl et al. 2020; Lascaratos et al. 2015). Together with current results that indicate high levels of HDL3 apolipoproteins and a more modest immune response in OHT patients compared to NTG patients, we suggest that a tightly controlled inflammatory system prevents excessive pro-inflammatory signals that otherwise may cause systemic damage including damage to the RGCs.

## Strengths and Limitations of the Study

The major challenge when investigating the immune response of human beings is that the level of inflammatory proteins is easily affected by multiple factors including age, diet, activity, medical conditions, and medication (Kondkar et al. 2018). This study was designed to limit the influence of diseases and medication. Moreover, great care was taken to select matching controls in terms of age, sex, and systemic conditions to the extent possible. We excluded participants with diseases or medication use that could affect measurements significantly. An influence of glaucomatous eyedrops on the regulation of circulating proteins can though not be completely ruled out (Arbabi et al. 2022; Haga et al. 2022). The duration of hypoxic intervention in the current study may have been too short to detect synthesis of proteins. In this context, the identified differential expression could relate to release, or lacking release, of pre-synthesized cytokines. The study may thus benefit from prolonging the hypoxia or recovery period. That would though be unethical since the duration of hypoxia is already similar or longer compared to other studies with a similar setup (Easton et al. 1985; Johansson et al. 2014; Petersen and Bek 2015; Vestergaard et al. 2016). Increasing the number of participants in baseline studies would improve the power to confirm findings in a larger cohort.

We believe that glaucoma is a manifestation of systemic disease and repsonses in the eye, due to the high demand for nutrients and protection in the unmyelinated RGCs. A circulating sample was assessed, to investigate the systemic inflammatory status. This strategy was expected to provide a better indication of the extension of inflammatory processes in glaucoma. We acknowledge that systemic levels of proteins may not reflect the retinal condition, and that cytokines in plasma are heavily diluted. Findings in previous studies, performing proteomics on aqueous humor samples, overlap extensively with proteins identified in the present study (Adav et al. 2018; Chowdhury et al. 2010; Kodeboyina et al. 2020; Lee et al. 2022; Liu et al. 2021), supporting the interaction between circulating plasma and aqueous humor levels. Aqueous humor samples offer a sample closer to the site of tissue degeneration in glaucoma. The total protein concentration in aqueous humor is though about five times lower compared to plasma, which could make it difficult to detect changes in low-abundance proteins like cytokines. To obtain aqueous humor, an eye surgery must be performed, which would demand the use of cataract patients as controls and make intervention impossible. Moreover, it would complicate the implementation of a biomarker in the future. Proteomic profiling in the current study was not intended for the discovery of a diagnostic biomarker, but can be useful to discover molecular mechanisms of glaucoma and even potential protein targets for medical intervention. These preliminary results are currently being verified in bigger populations in our research group.

## Conclusion

In conclusion, we applied proteomic and cytokine profiling to identify differentially expressed proteins in plasma from patients with NTG and OHT compared to controls. Our study indicates that NTG and OHT is associated with changes in circulating levels of immune- and hemostasis-related proteins. We suggest that the immune response may contribute to neurodegeneration in NTG. We suggest that OHT patients modulate their immune system in response to stress, to avoid excessive inflammatory proteins with potential harmful effects. Thus, we found that patients with OHT have specifically high levels of apolipoproteins, components of HDL3, that possess anti-inflammatory and antioxidant properties. Together with our previously published results that indicate a high antioxidant capacity and utilization of lipid metabolites and mediators, the current results suggest a tolerance towards development of glaucoma in OHT patients through limitation of inflammation and oxidative stress.

## Supplementary Information

Below is the link to the electronic supplementary material.

Supplementary file1 (DOCX 359 kb)

## Data Availability

The datasets generated during and/or analysed during the current study are not publicly available due to information that could compromise the privacy of research participants but are available from the corresponding author on reasonable request.
