# Correction: Proteomic and Cytokine Profiling in Plasma from Patients with Normal-Tension Glaucoma and Ocular Hypertension

**DOI:** 10.1007/s10571-024-01500-6

**Published:** 2024-10-04

**Authors:** Mia Langbøl, Arevak Saruhanian, Sarkis Saruhanian, Daniel Tiedemann, Thisayini Baskaran, Rupali Vohra, Amalie Santaolalla Rives, José Moreira, Verena Prokosch, Hanhan Liu, Jan-Wilm Lackmann, Stefan Müller, Claus Henrik Nielsen, Miriam Kolko, Jens Rovelt

**Affiliations:** 1 Department of Drug Design and Pharmacology, University of Copenhagen, Jagtvej 160, Building 22, 2100 Copenhagen Ø, Denmark; 2 Department of Veterinary & Animal Sciences, University of Copenhagen, Frederiksberg, Denmark; 3 Department of Ophthalmology, Copenhagen University Hospital, Rigshospitalet-Glostrup, Glostrup, Denmark; 4 Department of Ophthalmology, Faculty of Medicine and University Hospital of Cologne, University of Cologne, 50937 Cologne, Germany; 5 CECAD/CMMC Proteomics Facility, CECAD Research Center, University of Cologne, Cologne, Germany; 6 Institute for Inflammation Research, Center for Rheumatology and Spine Diseases, Rigshospitalet, Copenhagen University Hospital, 2200 Copenhagen, Denmark; 7 Department of Odontology, Faculty of Health and Medical Sciences, University of Copenhagen, 2200 Copenhagen, Denmark

**Correction to: Cellular and Molecular Neurobiology** 10.1007/s10571-024-01492-3

The original version of this article unfortunately missed to include one author in the author group.

The author José Moreira affiliated to Department of Drug Design and Pharmacology, University of Copenhagen, Jagtvej 160, Building 22, 2100 Copenhagen Ø, Denmark should be included as eight author of this article and so the Author Contributions section should be as follows:

