# Supplementary material for: Proteomic and Cytokine Profiling in Plasma from Patients with Normal-Tension Glaucoma and Ocular Hypertension
Source: Cell Mol Neurobiol. 2024 Aug 16;44:59. doi: 10.1007/s10571-024-01492-3 (PMC11329415; doi:10.1007/s10571-024-01492-3)
Supplement: Supplementary file 1 — Supplementary file1 (DOCX 359 kb) [file 10571_2024_1492_MOESM1_ESM.docx]

Supplementary information

Article title: Proteomic and Cytokine Profiling in Plasma from Patients with Normal-Tension Glaucoma and Ocular Hypertension

Journal name: Cellular and Molecular Neurobiology

Author names: Mia Langbøl ^1^, Arevak Saruhanian ^1^, Sarkis Saruhanian ^1,2^ Daniel Tiedemann ^1,3^, Thisayini Baskaran ^1^, Rupali Vohra ^1,3^, Amalie Santaolalla Rives ^1^, Verena Prokosch ^4^, Hanhan Liu ^4^, Jan-Wilm Lackmann ^5^, Stefan Müller ^5^, Claus H Nielsen ^6,7^, Miriam Kolko ^1,3^, and Jens Rovelt ^1^

Corresponding author:

Mia Langbøl Hjerrild, PhD

Department of Drug Design and Pharmacology, University of Copenhagen, Denmark

Email: mia.langboel@sund.ku.dk


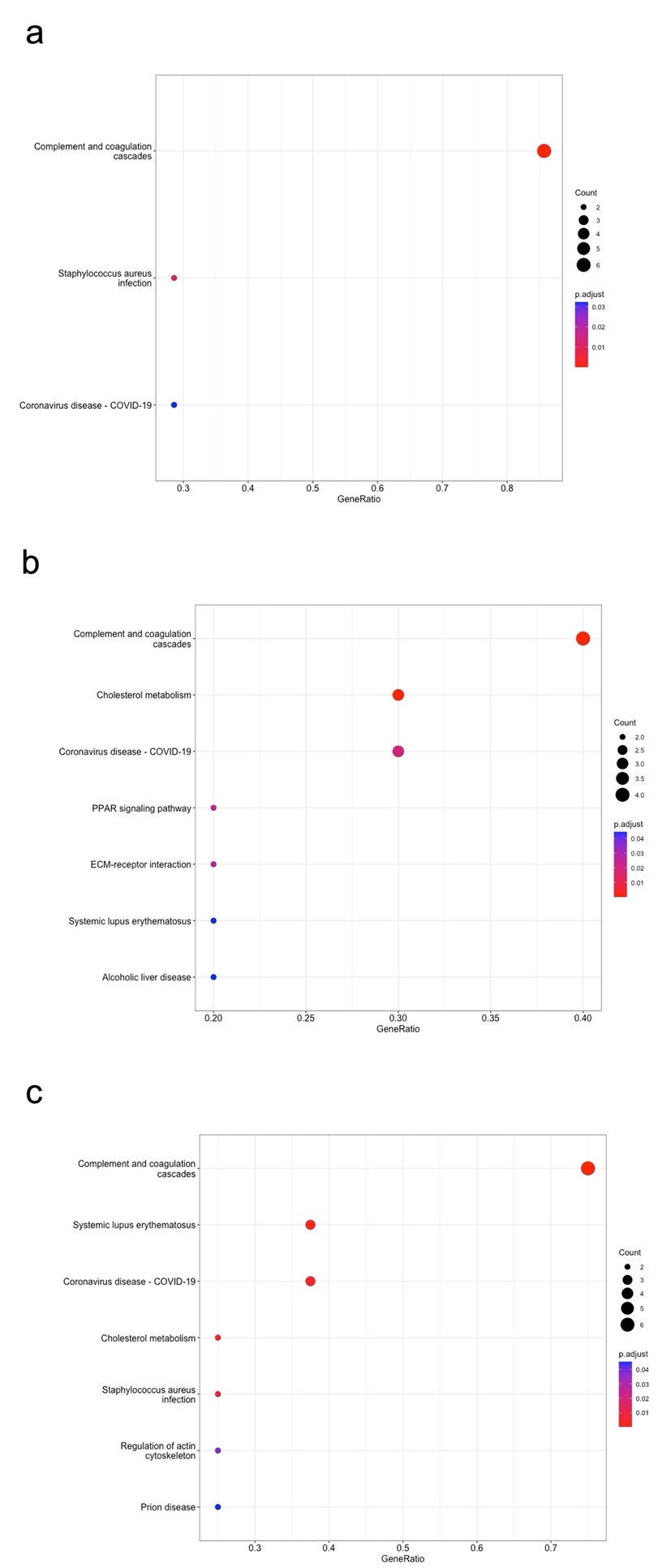


**Fig. S1** Differentially expressed proteins in plasma from patients with NTG compared to controls (**a**), patients with OHT compared to controls (**b**), and patients with NTG compared to patients with OHT (**c**) analyzed by KEGG pathway analysis. Results are presented as dotplots. Pathways are listed to the left and the gene ratio is indicated on the x-axis which is also illustrated by the size of the dot. Dots are colored according to the adjusted *p*-value. Red indicates a low *p*-value and blue a high *p*-value within the level of significance


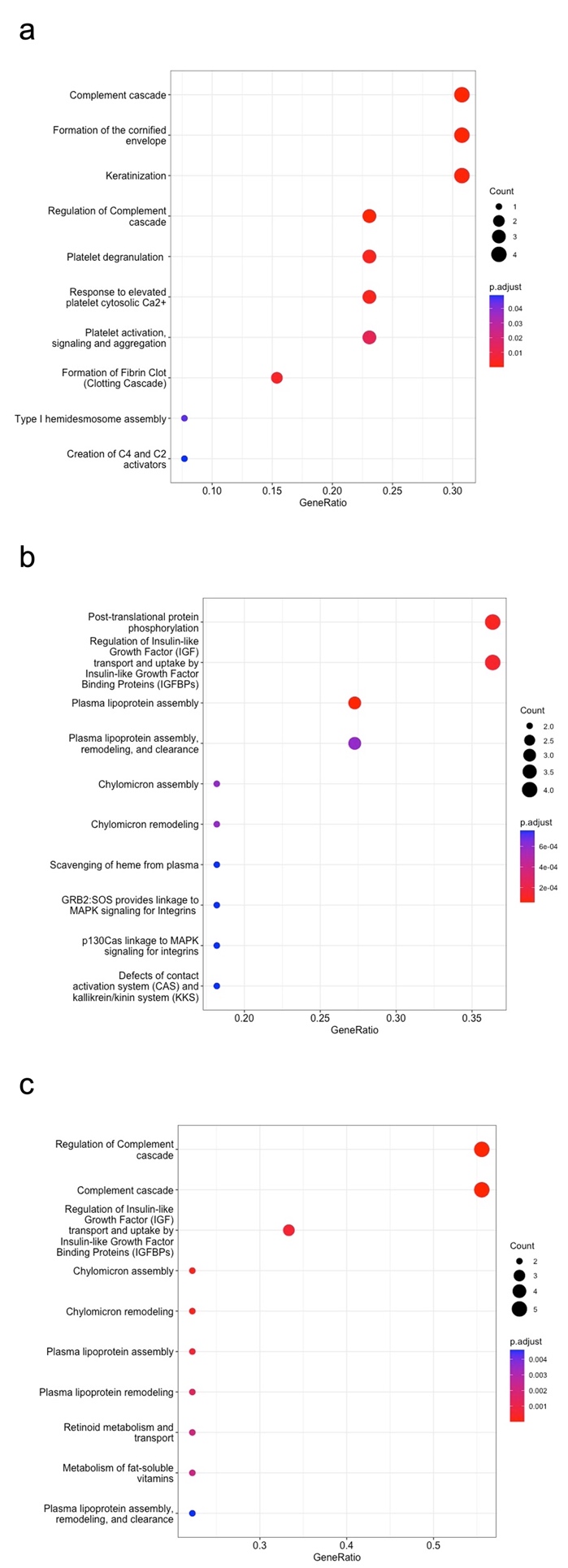


**Fig. S2** Differentially expressed proteins in plasma from patients with NTG compared to controls (**a**), patients with OHT compared to controls (**b**), and patients with NTG compared to patients with OHT (**c**) analyzed by Reactome pathway analysis. Results are presented as dotplots. Pathways are listed to the left and the gene ratio is indicated on the x-axis which is also illustrated by the size of the dot. Dots are colored according to the adjusted *p*-value. Red indicates a low *p*-value and blue a high *p*-value within the level of significance
